# Sensitivity to Environmental Stress and Adversity and Lung Cancer

**DOI:** 10.1001/jamanetworkopen.2024.57079

**Published:** 2025-01-29

**Authors:** Yingxi Chen, Qing Lan, Jing Yu, Devika Godbole, Jinyoung Byun, Christopher I. Amos, Haoyu Zhang

**Affiliations:** 1 Division of Cancer Epidemiology and Genetics, National Cancer Institute, National Institutes of Health, Rockville, Maryland; 2 Division of Population Health Research, Eunice Kennedy Shriver, National Institute of Child Health and Human Development, National Institutes of Health, Bethesda, Maryland; 3 Institute for Clinical and Translational Research, Department of Medicine, Baylor College of Medicine, Houston, Texas

## Abstract

**Importance:**

Sensitivity to environmental stress and adversity may influence lung cancer risk, highlighting a critical link between psychosocial factors and cancer etiology.

**Objective:**

To evaluate whether genetically estimated sensitivity to environmental stress and adversity is associated with lung cancer risk.

**Design, Setting, and Participants:**

Data were obtained from a genome-wide association study identifying 37 independent genetic variants strongly associated with sensitivity to environmental stress and adversity and a cross-ancestry genome-wide meta-analysis from the International Lung Cancer Consortium. Data were extracted between October 2023 and January 2024 and analyzed between February 2024 and June 2024.

**Exposures:**

Genetically estimated sensitivity to environmental stress and adversity.

**Main Outcomes and Measures:**

The main outcome was lung cancer risk, and odds ratios and 95% CIs were used to assess the association between sensitivity to environmental stress and adversity with lung cancer risk. This genetic association study used a 2-sample Mendelian randomization (MR) to estimate the association between genetically estimated sensitivity to environmental stress and adversity and lung cancer risk across different histologic types.

**Results:**

Using data from 351 827 individuals from the UK Biobank and a cross-ancestry genome-wide analysis of 61 047 lung cancer cases and 947 237 controls from the International Lung Cancer Consortium, sensitivity to environmental stress and adversity was significantly associated with an increased risk of lung cancer among individuals of European ancestry (OR, 1.49; 95% CI, 1.13-1.98; *P* = .005) and in the cross-ancestry analysis (OR, 1.45; 95% CI, 1.13-1.85; *P* = .004). There were heterogeneities in the observed associations across histologic subtypes among different population ancestries. Among individuals of European ancestry, a significant association was noted among squamous cell carcinoma (OR, 1.69; 95% CI, 1.06-2.70; *P* = .03). Among individuals of East Asian ancestry, an increased risk was observed for adenocarcinoma (OR, 2.04; 95% CI, 1.00-4.18; *P* = .05). Associations were not observed across any of the 3 histologic subtypes among individuals of African ancestry.

**Conclusions and Relevance:**

In this genetic association study, sensitivity to environmental stress and adversity was associated with lung cancer risk. These results underscore the necessity for further research into the nuanced association between psychosocial stress and cancer risk, with a particular focus on diverse populations.

## Key Points

**Question:**

Is genetically estimated sensitivity to environmental stress and adversity associated with the risk of lung cancer?

**Findings:**

In this genetic association study of 351 827 individuals from UK Biobank and a cross-ancestry genome-wide analysis of 61 047 lung cancer cases and 947 237 controls from the International Lung Cancer Consortium, sensitivity to environmental stress and adversity was significantly associated with a 49% increased risk of lung cancer in individuals of European ancestry and a 45% increased risk in the cross-ancestry analysis.

**Meaning:**

These findings suggest support for a potential causal link between sensitivity to environmental stress and adversity and the risk of lung cancer.

## Teaser

This genetic association study examines genetically estimated sensitivity to environmental stress and adversity among individuals with and without lung cancer.

## Introduction

Sensitivity to environmental stress and adversity is a highly heritable personality trait with significant effects on physical and mental health across the lifespan.^1^ Stress, arising from adverse external or internal forces, triggers the release of stress hormones, such as epinephrine and norepinephrine, affecting metabolism and other vital physiological functions.^2^ Environmental adversity can induce epigenetic changes, such as DNA methylation, altering the functioning of genes involved in stress responses, immune function, and inflammation.^3^ Moreover, chronic exposure to stress and adversity can lead to low-grade systemic inflammation, promoting tissue damage and dysregulating immune function.^4^ Consequently, prolonged exposure to stress and environmental adversity can lead to various health conditions, potentially increasing cancer risk, including lung cancer.^5^

In 2022, global lung cancer incidence varied widely with the highest rates observed in Central and Eastern Europe and Eastern Asia and the lowest in sub-Saharan Africa (except South Africa).^6^ Gender and regional differences are notable, with men experiencing the highest lung cancer incidence rates in Central and Eastern Europe, Southern Europe, Western Asia, and Melanesia (56 to 68 men per 100 000), while women have the highest rates in the United States, Canada, China, the Netherlands, and Denmark (30 to 35 women per 100 000).^6^ Historically, smoking patterns have driven these trends, with men in high-income countries adopting and quitting smoking earlier than women. Consequently, lung cancer death rates among men have declined since the late 1980s in regions such as North America, Northern and Western Europe, and Australia, while women death rates have plateaued or continue to rise due to delayed reductions in smoking prevalence. Other risk factors, such as environmental hazards and genetic susceptibility, also play important roles.^7,8,9^ While stress has been related to various adverse health outcomes, its association with lung cancer remains poorly understood. A meta-analysis of 16 observational studies reported a significant association between stress and lung cancer incidence.^5^ Despite this, the causal relationship remains uncertain, and it is unclear whether this association holds consistent across different histologic types of lung cancer.

In scenarios where randomized trials are not feasible, Mendelian randomization (MR) analysis offers a compelling alternative by using human genetic data.^10^ MR leverages naturally occurring genetic variants as unbiased proxies for exposures to make potential causal inference. Our study employs a 2-sample MR analysis, using single nucleotide variants (SNVs) as genetic instruments. Leveraging data from the largest multiancestry genome-wide association studies (GWAS) of lung cancer from the International Lung Cancer Consortium (ILCCO)^11^ and a GWAS published by Nagel et al^1^ on sensitivity to environmental stress and adversity using the UK Biobank, we aim to examine the causal association of sensitivity to environmental stress and adversity in relation to lung cancer risk. This analysis may provide novel insights to enhance our understanding of the etiology and biology of psychosocial stress in relation to lung cancer risk.

## Methods

This study follows the Strengthening the Reporting of Genetic Association Studies (STREGA) reporting guideline. Cohorts participating in the UK Biobank and the GWAS Lung Cancer Consortium received ethics approval from local institutional review boards and informed consent from all participants.

### Data Acquisition

We obtained summary-level statistic data for genetic instruments from the GWAS of sensitivity to environmental stress and adversity using data collected from individuals of European descent from the UK Biobank.^1^ Sensitivity to environmental stress and adversity was measured through questions assessing emotional reactivity, such as, “Are your feelings easily hurt?” “Do you worry too long after an embarrassing experience?” and “Are you often troubled by feelings of guilt?” These items, which were grouped based on their shared theme, define sensitivity to environmental stress and adversity as a distinct genetic cluster.^1^ We obtained the summary-level statistic data for lung cancer risk, both overall and across histologic subtypes, from a comprehensive multiancestry GWAS from 12 studies of diverse ancestry populations from the ILCCO published by Byun et al.^11^ The cohort predominantly consisted of individuals of European ancestry (74%), followed by those of East Asian (18%) and African ancestry (8%).^11^ The study specifically focused on histological subtypes of lung cancer, including small cell lung cancer, squamous cell carcinoma, and lung adenocarcinoma.

This study analyzed data from individuals of African ancestry, East Asian ancestry, and European ancestry. These populations were included to investigate potential differences in the association between sensitivity to environmental stress and adversity, and lung cancer risk across diverse populations. Such inclusion allows for a comprehensive assessment of factors that may vary by ancestry and influence susceptibility to lung cancer. Additionally, stratifying by ancestry helps ensure that findings are more generalizable and that disparities or unique patterns in lung cancer risk among different populations are appropriately captured and addressed.

### Instrumental Variables and Genetic Proxies for Exposure

For the current study, we selected 4463 genome-wide significant SNVs (*P* < 5 × 10^−8^) associated with sensitivity to environmental stress and adversity that were also present in the lung cancer outcome GWAS dataset.^1,11^ To maintain independence among genetic proxies, we then performed linkage disequilibrium (LD) clumping with a threshold of *r^2^* = 0.01, within a 1000 kb window using genotype data from European individuals of the 1000 Genomes Project (Phase 3) as reference panel,^12^ using PLINK version 1.9.^13^ This process yielded 38 SNVs. Subsequently, 1 SNV (rs7938812), known to be associated with smoking behavior,^14^ was excluded to prevent potential confounding. The remaining 37 SNVs do not have known associations with risk or carcinogenic factors associated with lung cancer, and thus, were included in the MR analysis (Table 1 and eTable 1 in Supplement 1).

**Table 1.** Candidate Genetic Instruments of Sensitivity to Environmental Stress and Adversity

Selection criteria	Participants and genetic instruments, No.			
	Ancestry of participants	Candidate genetic instruments	Genetic instruments after LD clumping	Final genetic instruments used in the analysis
*P* < 5 × 10^−8^	European, UK Biobank, 351 827	4463	38	37

Abbreviation: LD, linkage disequilibrium.

### Statistical Analysis

We conducted a 2-sample MR analysis primarily using the robust adjusted profile score (MR-RAPS) method to estimate the odds ratio (OR) and 95% CI for the association between sensitivity to environmental stress and adversity with lung cancer risk, both overall and across histologic subtypes.^15^ We conducted sensitivity analysis using 2 additional MR methods, debiased inverse-variance weighted (dIVW) and MR median, to evaluate robustness of the results. To assess if the association differed by population ancestry and to provide cross-ancestry estimates, we performed meta-analysis using random effects model. All statistical analyses were performed in R version 4.3.1 (R Project for Statistical Computing) with the packages MendelianRandomization, mr.raps, and meta. A 2-tailed *P* < .05 was considered statistically significant. Summary statistics are available at the following links: sensitivity to environmental stress and adversity^16^ and lung cancer.^17^

## Results

### Association With Overall Lung Cancer Risk

In this genetic association study of 351 827 individuals from the UK Biobank and a cross-ancestry genome-wide analysis of 61 047 lung cancer cases and 947 237 controls from the International Lung Cancer Consortium, sensitivity to environmental stress and adversity was significantly associated with an increased risk of lung cancer in individuals of European ancestry (OR, 1.49; 95% CI, 1.13-1.98; *P* = .005) and in the cross-ancestry analysis (OR, 1.45; 95% CI, 1.13-1.85; *P* = .004) (Table 2 and Figure). Nonetheless, for individuals of African and East Asian ancestry, these associations did not reach statistical significance. Among individuals of African ancestry, the OR was 1.33 (95% CI, 0.50-3.55; *P* = .57), while among those of East Asian ancestry, the OR was 1.26 (95% CI, 0.66-2.41; *P* = .48). Sensitivity analyses using the dIVW and MR-Median methods yielded consistent results overall (eTable 2 and eFigure 1 in Supplement 1).

**Table 2.** Mendelian Randomization Estimates of Stress on Lung Cancer and by Histologic Subtypes, Across Ancestry

Outcome by ancestry	No.		OR (95%CI)	*P* value^a^
	Cases	Controls		
European				
Lung cancer	26 683	25 278	1.49 (1.13-1.98)	.005
Squamous cell carcinoma	6107	23 173	1.69 (1.06-2.70)	.03
Adenocarcinoma	2267	23 173	1.20 (0.81-1.79)	.37
Small cell lung cancer	9791	23 173	1.73 (0.82-3.65)	.15
East Asian				
Lung cancer	7062	5372	1.26 (0.66-2.41)	.48
Squamous cell carcinoma	1292	5372	0.58 (0.18-1.80)	.34
Adenocarcinoma	4630	5372	2.04 (1.00-4.18)	.05
Small cell lung cancer	99	5372	0.04 (0.00-2.05)	.11
African				
Lung cancer	1987	3774	1.33 (0.50-3.55)	.57
Squamous cell carcinoma	451	3774	2.99 (0.53-17.00)	.22
Adenocarcinoma	844	3774	0.58 (0.15-2.22)	.43
Small cell lung cancer	116	3774	1.17 (0.04-31.45)	.93

Abbreviation: OR, odds ratio.

^a^ *P* value estimates are based on Mendelian randomization with summary statistics by using Robust Adjusted Profile Score (ie, MR-RAPS).^15^

**Figure.**
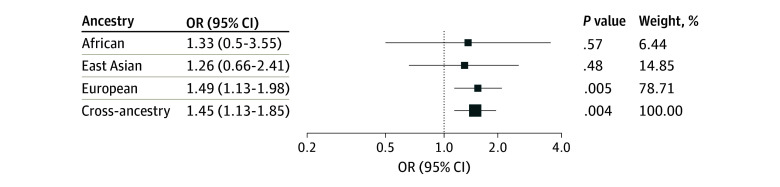
Summary of Results for Sensitivity to Environmental Stress and Adversity and Overall Lung Cancer Risk Results from Mendelian randomization with summary statistics by using Robust Adjusted Profile Score analysis.

### Association With Lung Cancer Risk by Histologic Subtype

We observed heterogeneity in the association between sensitivity to environmental stress and adversity and lung cancer risk across histologic subtypes among different population ancestries (Table 2 and eFigure 2 in Supplement 1). Among individuals of European ancestry, a significant association was noted among squamous cell carcinoma, with an OR of 1.69 (95% CI, 1.06-2.70; *P* = .03) (Table 2). However, associations with adenocarcinoma and small cell lung cancer did not reach statistical significance. Among individuals of East Asian ancestry, sensitivity to environmental stress and adversity was associated with an increased risk of adenocarcinoma (OR, 2.04; 95% CI, 1.00-4.18; *P* = .05). Notably, associations were not observed across any of the 3 histologic subtypes among individuals of African ancestry.

## Discussion

In this 2-sample MR analysis using GWAS summary data, we found a significant association of genetically estimated sensitivity to environmental stress and adversity in relation to lung cancer risk among individuals of European ancestry, as well as in a broader cross-ancestry analysis. Stress, as a physiological and psychological response to adverse conditions, is critically implicated in immune dysfunction that influence disease susceptibility.^18^ Prolonged exposure to stress is particularly concerning, as it can lead to enduring changes in emotional, physiological, and behavioral responses, thereby increasing individuals’ susceptibility to various health conditions.^18,19^ Those with increased sensitivity to environmental stress and adversity are likely exposed to chronic stress and hence heightened risk for worse health.

The association between stress and lung cancer is complex. Robust evidence suggests that stress can facilitate cancer progression by modulating key cancer hallmarks, as seen in animal studies and partially supported by clinical observations.^20^ A 2008 meta-analysis of 142 studies (mean sample size of 87 000) indicated that psychosocial stress estimates a modest increase in cancer incidence (HR, 1.06; 95% CI, 1.02-1.11; *P* = .005), largely driven by depression.^5^ Studies on work stress as a cancer risk factor have yielded mixed results, with 1 meta-analysis^21^ reporting null effects and another indicating elevated relative risks for lung and colorectal cancers.^22^ Despite the inconsistent effects observed in epidemiologic studies, animal studies suggest that stress can impact cancer progression by targeting stress-sensitive phases of tumor growth and metastasis.^20^ Unlike animal studies, epidemiologic studies and clinical trials on stress-reducing interventions typically do not focus on such stress-prone phases, which may explain the mixed results.

The current study revealed a significant association between sensitivity to environmental stress and adversity and an increased risk of lung cancer. Malignant development in humans is a prolonged process and subclinical cancer dormancy is common.^23^ Therefore, cancer risk may be elevated both through disease initiation and through escape from dormancy or accelerated progression to clinical manifestation. Animal studies have shown that stress factors can trigger tumor cells to escape dormancy.^24^ Further research is warranted to elucidate the underlying mechanisms driving the observed associations among humans. Moreover, our histologic subtype specific analysis showed heterogeneity across different population ancestries. The observed variations may reflect underlying genetic predisposition, environmental, and lifestyle differences, highlighting the complexity of lung cancer etiology. It also reflected the importance of considering both population diversity and histologic subtype when exploring lung cancer risk.

### Limitations

There are several limitations in the study. The genetic proxies were primarily associated with sensitivity to environmental stress and adversity, falling under the broader category of neuroticism—a psychological trait marked by a propensity toward emotions like guilt, embarrassment, and being easily hurt. Hence, these genetic markers may not fully capture the dynamic nature of sensitivity to all types of psychosocial stress. Additionally, the genetic instruments derived from populations of European ancestry. The relatively small sample sizes for individuals of African and East Asian ancestries constrained our statistical power, particularly in detecting associations across different histologic subtypes.

## Conclusions

In this genetic association study of 351 827 individuals from the UK Biobank and a cross-ancestry genome-wide analysis of 61 047 lung cancer cases and 947 237 controls, sensitivity to environmental stress and adversity were associated with lung cancer risk. Our findings suggest variability in this association across different lung cancer histologic subtypes and ancestries, pointing to the complex etiology and development of lung cancer. These results not only contribute to our understanding of lung cancer’s multifaceted nature but also underscore the necessity for further research into the nuanced association between psychosocial stress and cancer risk, with a particular focus on diverse populations.

## Notes

Supplement 1. **eTable 1. **Single-Nucleotide Variants Included in Genetic Instruments **eTable 2. **Sensitivity Analysis of Mendelian Randomization Estimates of Stress on Lung Cancer and by Histologic Subtypes, Across Ancestry **eFigure 1.** Mendelian Randomization Estimates of Stress on Lung Cancer, by Histologic Subtypes, Across Ancestry **eFigure 2.** Meta-Analysis of Mendelian Randomization Estimates (MR_RAPS) of Stress on Lung Cancer by Histologic Subtypes, Across Ancestry

Supplement 2.
Data Sharing Statement
