# Supplementary material for: Sensitivity to Environmental Stress and Adversity and Lung Cancer
Source: JAMA Netw Open. 2025 Jan 29;8(1):e2457079. doi: 10.1001/jamanetworkopen.2024.57079 (PMC11780474; doi:10.1001/jamanetworkopen.2024.57079)
Supplement: Supplement 1. — eTable 1. Single-Nucleotide Variants Included in Genetic Instruments eTable 2. Sensitivity Analysis of Mendelian Randomization Estimates of Stress on Lung Cancer and by Histologic Subtypes, Across Ancestry eFigure 1. Mendelian Randomization Estimates of Stress on Lung Cancer, by Histologic Subtypes, Across Ancestry eFigure 2. Meta-Analysis of Mendelian Randomization Estimates (MR_RAPS) of Stress on Lung Cancer by Histologic Subtypes, Across Ancestry [file jamanetwopen-e2457079-s001.pdf]

Supplemental Online Content

Chen Y, Lan Q, Yu J, et al. Sensitivity to environmental stress and adversity and lung cancer. *JAMA Netw Open*. 2025;8(1):e2457079. doi:10.1001/jamanetworkopen.2024.57079

eTable 1. Single-Nucleotide Variants Included in Genetic Instruments

eTable 2. Sensitivity Analysis of Mendelian Randomization Estimates of Stress on Lung Cancer and by Histologic Subtypes, Across Ancestry

eFigure 1. Mendelian Randomization Estimates of Stress on Lung Cancer, by Histologic Subtypes, Across Ancestry

eFigure 2. Meta-Analysis of Mendelian Randomization Estimates (MR_RAPS) of Stress on Lung Cancer by Histologic Subtypes, Across Ancestry

This supplemental material has been provided by the authors to give readers additional information about their work.

© 2025 Chen Y. *JAMA Network Open*.

eTable 1. Single-Nucleotide Variants Included in Genetic Instruments

SNP	ID	Effect allele Frequency	Beta	Gene	Reported Trait
rs10119773	9:23736400:A_G	0.463	-0.01576	ELAVL2	Neuroticism; feeling guilty; anxiety; depressive symptoms; sensitivity to environmental stress and adversity
rs11022762	11:13335926:C_T	0.3833	0.01662	BMAL1	Hematocrit; hemoglobin; sensitivity to environmental stress and adversity; red blood cell count; worry/vulnerability
rs11115146	12:82387729:A_G	0.1266	0.01948	NR	Sensitivity to environmental stress and adversity
rs11167957	5:146436523:C_T	0.3827	-0.01324	PPP2R2B	Sensitivity to environmental stress and adversity
rs11665070	18:35152563:A_G	0.3315	0.02149	MIR4318, CELF4	Depression; subjective wellbeing; educational attainment; sensitivity to environmental stress and adversity
rs11767715	7:39129941:C_T	0.1327	-0.02115	POU6F2	Feeling hurt; sensitivity to environmental stress and adversity
rs12525800	6:12035329:A_C	0.4954	-0.01311	NR	Sensitivity to environmental stress and adversity
rs13299873	9:4145545:A_G	0.2834	0.01637	NR	Sensitivity to environmental stress and adversity
rs1941356	18:31612400:C_T	0.361	0.01371	NR	Sensitivity to environmental stress and adversity
rs1978573	2:185459544:C_T	0.2983	-0.01454	MIR548AE1, ZNF804A	Sensitivity to environmental stress and adversity; feeling hurt
rs2072632	6:31921475:A_G	0.3047	0.01584	NELFE	Serum levels of protein SPAG11B (blood protein measurement); sensitivity to environmental stress and adversity
rs2157753	7:126472723:A_C	0.4938	0.01822	NR	Sensitivity to environmental stress and adversity
rs2191129	16:7667995:C_T	0.4821	-0.01541	NR	Sensitivity to environmental stress and adversity
rs2282040	9:98248328:A_G	0.09428	0.02858	PTCH1	Sensitivity to environmental stress and adversity

© 2025 Chen Y. *JAMA Network Open*.

rs2627019	2:148729050:C_T	0.435	0.01292	ORC4	Sensitivity to environmental stress and adversity
rs314289	6:105430934:C_T	0.4385	-0.01296	LIN28B	Sensitivity to environmental stress and adversity
rs3748400	16:87445839:C_T	0.2256	0.01571	ZCCHC14	Neuroticism; highest math class taken; height; sensitivity to environmental stress and adversity
rs3772882	3:81808602:A_C	0.3727	-0.01465	GBE1	Sensitivity to environmental stress and adversity; BMI
rs3816447	15:35231184:A_G	0.2691	-0.01532	NR	Sensitivity to environmental stress and adversity
rs391957	9:128004024:C_T	0.4066	-0.01506	HSPA5-DT	Sensitivity to environmental stress and adversity; anxiety
rs4245154	11:113388674:A_G	0.4342	-0.01912	TMPRSS5, DRD2	Sensitivity to environmental stress and adversity; feeling miserable; worry too long after an embarrassing experience; sociability score
rs4653218	1:37197319:C_T	0.42	0.01617	GRIK3, FTLP18	Depression; sensitivity to environmental stress and adversity
rs4766471	12:109853462:A_G	0.2503	0.01841	NR	Sensitivity to environmental stress and adversity
rs4791331	17:8932082:C_T	0.4633	-0.0139	NTN1	Sensitivity to environmental stress and adversity
rs4868748	5:164437147:A_G	0.2672	0.01453	LINC03000	Sensitivity to environmental stress and adversity
rs627387	11:88694072:C_T	0.4845	0.0137	NR	Sensitivity to environmental stress and adversity
rs6439649	3:136371691:G_T	0.3987	-0.01432	STAG1	Sensitivity to environmental stress and adversity; worry too long after an embarrassing experience
rs66530963	17:79097769:G_T	0.1431	-0.01925	AATK	Sensitivity to environmental stress and adversity; depressive symptoms; life satisfaction
rs6743916	2:58704449:A_G	0.2938	-0.01455	LINC01122	Sensitivity to environmental stress and adversity
rs6921256	6:27587518:C_T	0.3822	-0.01323	RPL8P1, RNU6-471P	Inguinal hernia; sensitivity to environmental stress and adversity
rs7294498	12:118759485:A_C	0.1606	0.01918	NR	Sensitivity to environmental stress and adversity

© 2025 Chen Y. *JAMA Network Open*.

rs7567451	2:157053380:G_T	0.2687	-0.0144	LINC01876	Worry; feeling worry; anxiety; sensitivity to environmental stress and adversity
rs761898	14:69480381:A_G	0.377	-0.01362	ACTN1-DT, RPS29P1	Feeling hurt; sensitivity to environmental stress and adversity; drinks per week
rs7936998	11:57639017:A_G	0.3269	0.01829	OR5BA1P, OR5AZ1P	Diet measurement; sensitivity to environmental stress and adversity
rs7938812	11:112911004:G_T	0.3874	0.01327	NCAM1	Smoking behavior; sensitivity to environmental stress and adversity
rs7939992	11:113229665:C_T	0.4837	-0.01434	NR	Sensitivity to environmental stress and adversity
rs8073146	17:43893751:A_G	0.2237	0.02621	NR	Sensitivity to environmental stress and adversity
rs9811585	3:35724596:G_T	0.3805	0.01418	ARPP21	Worry too long after an embarrassing experience; sensitivity to environmental stress and adversity

© 2025 Chen Y. *JAMA Network Open*.

eTable 2. Sensitivity analysis of Mendelian randomization estimates of stress on lung cancer and by histologic subtypes, across ancestry.

Population Ancestry	Outcome	dIVW		Median	
		OR (95%CI)	*P*-value	OR (95%CI)	P-value
European	Lung cancer	1.48 (0.98-2.22)	0.061	1.82 (1.89-2.80)	0.009
	Squamous cell carcinoma	1.68 (0.95-2.98)	0.076	1.79 (0.90-3.57)	0.098
	Adenocarcinoma	1.20 (0.69-2.07)	0.522	1.37 (0.76-2.48)	0.297
	Small cell lung cancer	1.73 (0.78-3.80)	0.178	1.79 (0.62-5.14)	0.281
East Asian	Lung cancer	1.26 (0.66-2.41)	0.477	0.85 (0.36-2.04)	0.722
	Squamous cell carcinoma	0.57 (0.18-1.78)	0.336	0.48 (0.10-2.21)	0.345
	Adenocarcinoma	2.05 (1.00-4.19)	0.049	1.59 (0.61-4.16)	0.342
	Small cell lung cancer	0.04 (0.00-1.96)	0.105	0.03(0.00-5.21)	0.177
African	Lung cancer	1.33 (0.50-3.54)	0.565	2.02 (0.56-7.24)	0.282
	Squamous cell carcinoma	2.98 (0.52-17.01)	0.219	1.62 (0.14-19.36)	0.704
	Adenocarcinoma	0.58 (0.15-2.21)	0.425	0.66 (0.10-4.35)	0.664
	Small cell lung cancer	1.17 (0.02-76.03)	0.942	0.66 (0.001-88.03)	0.868

© 2025 Chen Y. *JAMA Network Open*.

eFigure 1. Mendelian randomization estimates of stress on lung cancer, by histologic subtypes, across ancestry.

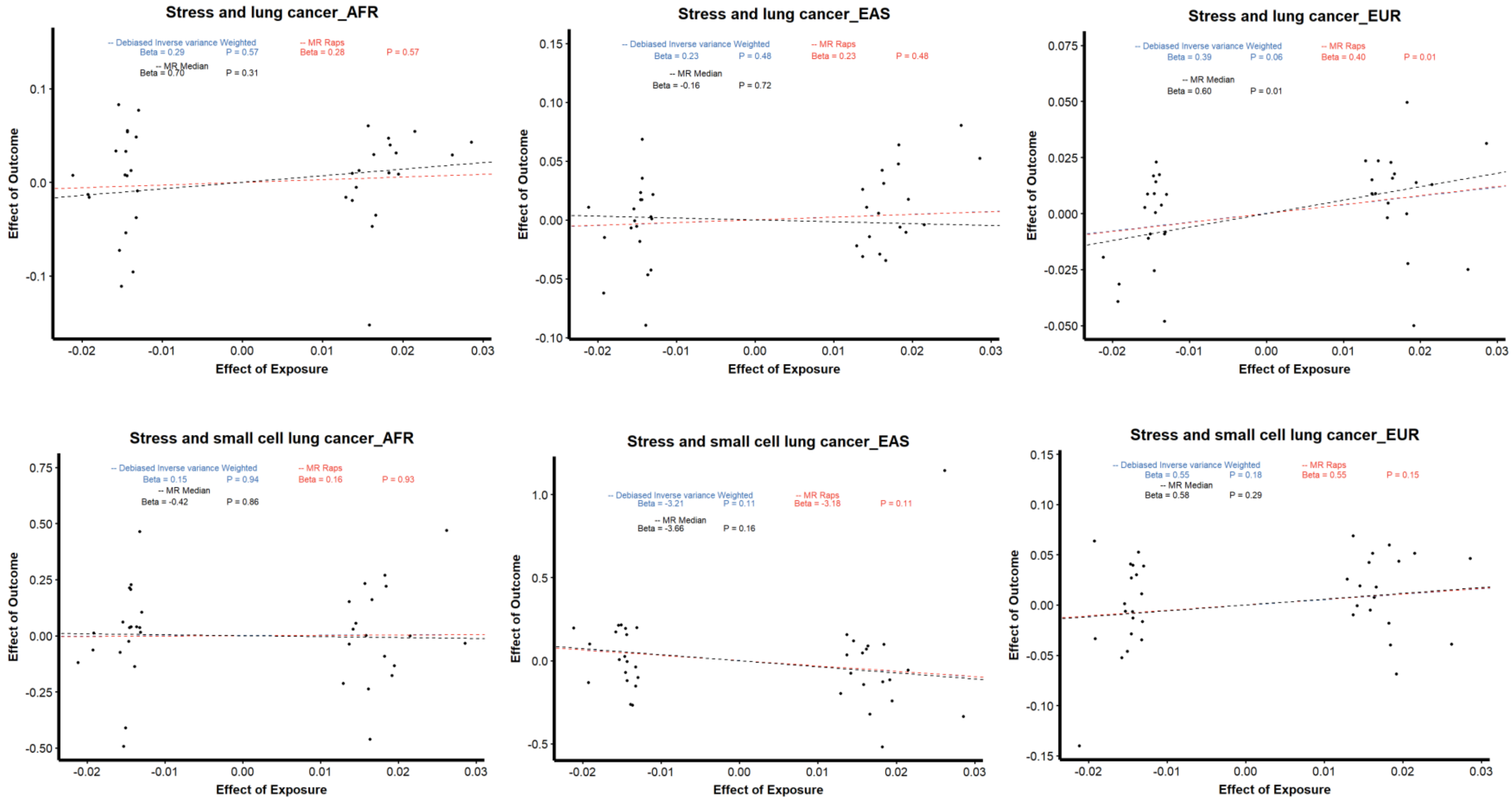

© 2025 Chen Y. *JAMA Network Open*.

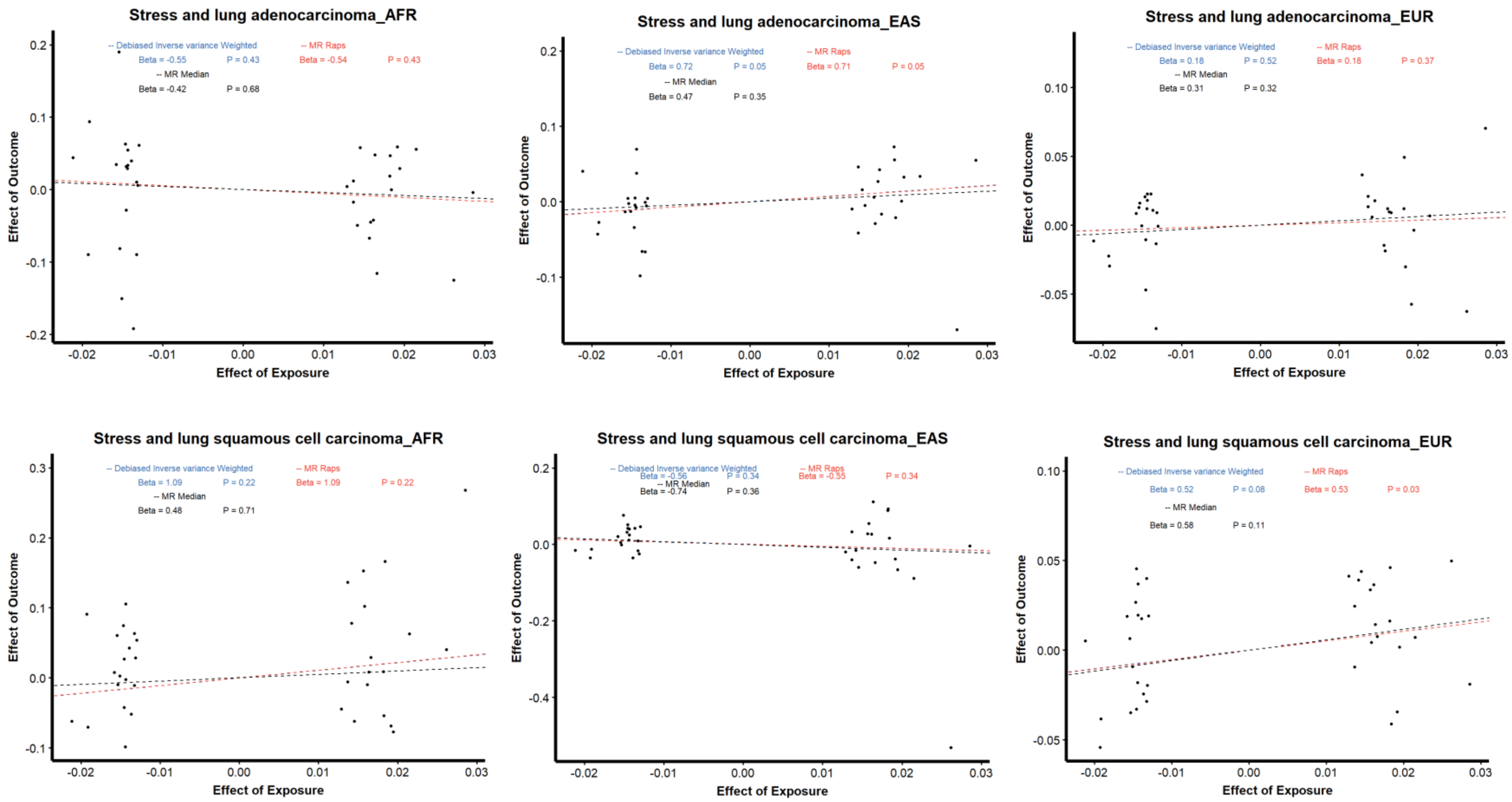

© 2025 Chen Y. *JAMA Network Open*.

eFigure 2. Meta-analysis of Mendelian randomization estimates (MR_RAPS) of stress on lung cancer by histologic subtypes, across ancestry.

2a. Small cell lung cancer

Study	OR (95% CI)	P-value	Weight
**African**	1.17 (95% CI: 0.04 - 31.45)	0.9255	4.73%
**East Asian**	0.04 (95% CI: 0.00 - 2.05)	0.1099	3.36%
**European**	1.73 (95% CI: 0.82 - 3.65)	0.1512	91.91%
**Meta-Analysis**	0.89 (95% CI: 0.16 - 5.09)	0.8963	100.00%

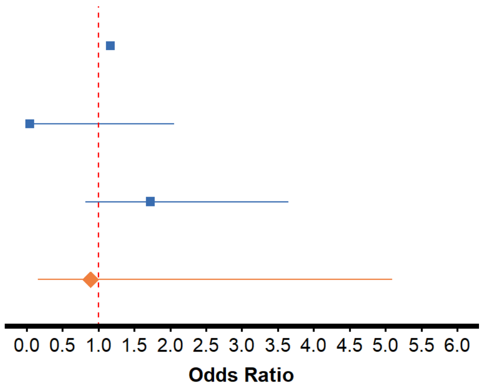

2b. Adenocarcinoma

Study	OR (95% CI)	P-value	Weight
**African**	0.58 (95% CI: 0.15 - 2.22)	0.4268	6.30%
**East Asian**	2.04 (95% CI: 1.00 - 4.18)	0.0513	22.04%
**European**	1.20 (95% CI: 0.81 - 1.79)	0.3681	71.66%
**Meta-Analysis**	1.30 (95% CI: 0.86 - 1.98)	0.2181	100.00%

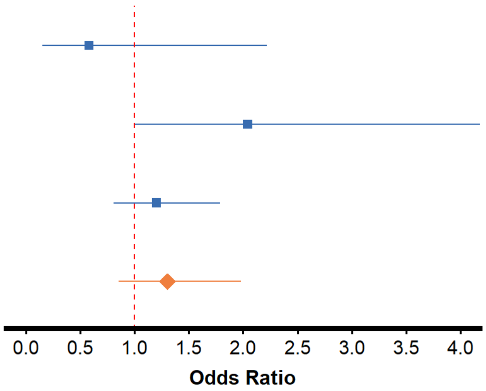

2c. Squamous cell carcinoma

Study	OR (95% CI)	P-value	Weight
**African**	2.99 (95% CI: 0.53 - 17.00)	0.2172	5.81%
**East Asian**	0.58 (95% CI: 0.18 - 1.80)	0.3419	13.53%
**European**	1.69 (95% CI: 1.06 - 2.70)	0.0272	80.65%
**Meta-Analysis**	1.37 (95% CI: 0.64 - 2.92)	0.4172	100.00%

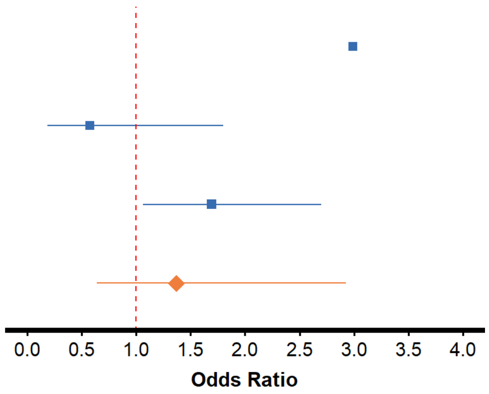

© 2025 Chen Y. *JAMA Network Open*.
