# Supplementary material for: Sensitivity to Environmental Stress and Adversity and Lung Cancer
Source: JAMA Netw Open. 2025 Jan 29;8(1):e2457079. doi: 10.1001/jamanetworkopen.2024.57079 (PMC11780474; doi:10.1001/jamanetworkopen.2024.57079)
Supplement: Supplement 2. — Data Sharing Statement [file jamanetwopen-e2457079-s002.pdf]

Data Sharing Statement

Data
Data available: Yes
Data types: Deidentified participant data
How to access data: Deidentified participant data
When available: With publication

Supporting Documents
Document types: None

Additional Information
Who can access the data: The data used for this analysis can be made available upon reasonable request to Mats Nagel at m.nagel@vu.nl; and Chris Amos at chris.amos@bcm.edu
Types of analyses: For research purpose only
Mechanisms of data availability: With investigator support
